# Molecular Techniques for the Detection and Differentiation of Host and Parasitoid Species and the Implications for Fruit Fly Management

**DOI:** 10.3390/insects3030763

**Published:** 2012-08-14

**Authors:** Cheryl Jenkins, Toni A. Chapman, Jessica L. Micallef, Olivia L. Reynolds

**Affiliations:** Elizabeth Macarthur Agricultural Institute, NSW Department of Primary Industries, Woodbridge Road, Menangle, NSW 2568, Australia; E-Mails: toni.chapman@dpi.nsw.gov.au (T.A.C.); jess.smart@dpi.nsw.gov.au (J.L.M.); olivia.reynolds@dpi.nsw.gov.au (O.L.R.)

**Keywords:** Diptera, Tephritidae, parasitoid, biological control, DNA barcode, PCR, microsatellites

## Abstract

Parasitoid detection and identification is a necessary step in the development and implementation of fruit fly biological control strategies employing parasitoid augmentive release. In recent years, DNA-based methods have been used to identify natural enemies of pest species where morphological differentiation is problematic. Molecular techniques also offer a considerable advantage over traditional morphological methods of fruit fly and parasitoid discrimination as well as within-host parasitoid identification, which currently relies on dissection of immature parasitoids from the host, or lengthy and labour-intensive rearing methods. Here we review recent research focusing on the use of molecular strategies for fruit fly and parasitoid detection and differentiation and discuss the implications of these studies on fruit fly management.

## 1. Introduction

Fruit flies (Diptera: Tephritidae) are one of the most significant group of pests affecting fruit and fruiting vegetables worldwide. This family includes over 4,000 species, approximately 1,400, of which attack soft fruits [1]. The four major groups of fruit pest tephritids, *Ceratitis*, *Bactrocera* (and *Dacus*), *Anastrepha* and *Rhagoletis*, include several of the key pest species that affect market access [1].

In the early 20th century parasitic Hymenoptera were first identified and utilised for the management of tephritid pests with attempts made to locate natural enemies for the Mediterranean fruit fly, *Ceratitis capitata* (Wiedemann) in Western Australia [2] and the olive fly, *Bactrocera oleae* (Gmelin) in Italy [3]. The subsequent successful introduction and establishment of several parasitoid species from Africa into Hawaii for the control of *C. capitata* in 1913 [3] has resulted in a focus on tephritid parasitoid wasps ever since. Classical introductions of parasitoid wasps were the most common form of biological control, however with the establishment of the introduced species [4], augmentative and inundative releases [5], often paired with releases of sterile flies [6,7] are now common [8].

Over 100 species of Hymenoptera (Braconidae) have been reared from fruit-infesting tephritids from nearly every continent, with the majority from the subfamily Opiinae [1,9,10,11]. Parasitoids from several genera within this subfamily including *Diachasmimorpha*, *Fopius*, *Aganaspis* and *Psyttalia* (Hymenoptera: Braconidae) are thought to be the most promising or are currently employed in the biological control of fruit fly pests [12,13,14,15]. These parasitoids are all solitary, koinobiont endoparasitoids, which oviposit in the host egg, larval or pupal stage of the fly, completing their life cycle and eclosing as adult parasitoids from the host puparium [13]. There are incidences where oviposition by a parasitoid occurs and a fruit fly still emerges, the cause of which is believed to be encapsulation, where the immune defense of the host larvae overcomes the foreign egg [16,17]. Nonetheless, parasitoids are target-specific and environmentally-friendly, and when used appropriately and on an area-wide basis [18], they are effective tools for the sustainable control of fruit fly pests [19].

Since early last century, several species of parasitoids have been introduced into non-endemic countries for the control of local fruit fly pests. For example, *Fopius ceratitivorus* (Wharton) and *Diachasmimorpha kraussii* (Fullaway) were introduced into to Israel to control *C. capitata* [20] and several species including *Diachasmimorpha longicaudata* (Ashmead) and *Psyttalia concolor* (Silvestri) were introduced into Florida, United States of America for the control of *Anastrepha suspensa* (Loew) [21]. Further, several species, have been used successfully as part of an augmentative or inundative release program including *Diachasmimorpha longicaudata* for the control of *Anastrepha suspensa* in Florida [22] and of *C. capitata* in Mexico and Guatemala [23], and *Diachasmimorpha tryoni* (Cameron) for the control of *C. capitata* on Maui, Hawaii [7,24] and along the Mexican/Guatemalan border [25]. 

The inclusion of parasitoids into integrated pest management programs has increased in recent times due to significant advances in rearing techniques [26], diets [27,28] and intensive research into parasitoid biology, ecology and behaviour [29,30,31]. However, a clear understanding of parasitoid host specificity and preference, as well as knowledge of the genetic diversity within a given fruit fly population, are among the most critical factors in determining the success of fruit fly control programs involving the sterile insect technique (SIT) and augmentative release [32]. With the increasing use of parasitoid wasps for biological control of tephritids, efficient and effective identification, differentiation and monitoring tools are needed for parasitoids and their hosts alike. 

Biological control of arthropod pests, including tephritids, has traditionally relied on phenotypic and morphological methods for characterising species of hosts and their parasitoids [33,34]. In more recent years, molecular techniques have become important tools in insect pest management, complementing and in some cases superseding morphological methods for accurate identification of these organisms [35,36,37,38]. Indeed, one advantage of molecular techniques over phenotypic methods is that they bypass the need for the specialist entomological knowledge required for morphological species differentiation and can be performed in a variety of laboratory settings [39]. Furthermore, molecular methods can overcome difficulties in differentiating morphologically similar species, larval forms or members of cryptic species complexes [40,41]. Therefore, implementation of robust molecular diagnostic techniques should lead to increased success of fruit fly biological control programs through enhanced understanding of host and parasitoid population genetics and improved matching of parasitoid species to their preferred host/s. In this paper, we review the status of research into the development of molecular strategies for host and parasitoid detection and differentiation and discuss the implications of these studies for the biological control of tephritid fruit flies.

## 2. Molecular Methods Employed in Biological Control of Arthropods

### 2.1. DNA-Barcoding

DNA barcoding involves the PCR amplification and sequencing of a key genetic marker from a given specimen and is one of the most widely used molecular techniques employed in the study and management of arthropod pests and their parasitoids [37]. The technique relies on the availability of a large database of sequence orthologs for comparison, and when identifying an unknown sample, it requires prior knowledge of the relevant sequence of the species in question. In this context, DNA barcoding can be used as a species-specific diagnostic [42] and has been applied relatively successfully to species identification [43], differentiation [44,45], and the study of geographical variation in host-parasitoid interactions [46]. DNA barcodes are also widely used for taxonomic analyses [13,47,48]. Indeed, it is prudent to conduct a phylogenetic analysis of key genetic markers within a species to determine intra-species relationships, as well as evolutionary relationships with other taxa, prior to attempting DNA barcoding as a species-specific diagnostic [40]. 

DNA barcoding typically refers to the amplification and sequencing of the 5' portion of mitochondrial cytochrome oxidase I (COI) gene. The development of a set of PCR primers [49] capable of amplifying the COI barcode region from a diverse array of animals has ensured the widespread use of this region for species discrimination, including arthropods [42]. However, alternative molecular markers may be sequenced depending on the level of taxonomic discrimination required [13,50,51]. Thus, genetic markers with a high level of sequence variability such as the ribosomal DNA intergenic transcribed spacer regions (ITS-1 and ITS-2) may be most useful for differentiating variants within a species complex [52,53], while highly conserved markers, such as the nuclear 28S ribosomal rRNA (28S rRNA) gene (particularly the more variable D2 loop region) may be more appropriate for discriminating between species or more distantly related taxa [13]. The COI barcode region can be considered to be in a “goldilocks zone” for taxonomic utility [54]; it displays a relatively high level of sequence conservation, but is more variable than 28S rDNA (and other ribosomal rDNAs) and is considerably less variable than the ITS regions and many mitochondrial genetic markers. As such, it is useful for the discrimination of both closely related and more distantly related taxa [42,55,56].

The technique of COI barcoding has become more prevalent in the study of tephritids and their parasitoids in recent years, particularly in an attempt to resolve relationships within species complexes. Indeed, the COI barcode region has been used to differentiate fruit fly parasitoids within the *Psyttalia* genus [13], the *Diachasmimorpha* genus [48] and tephritids of the *Bactrocera dorsalis* (Hendel) [43], *Bactrocera tryoni* (Froggatt) [44] and *Ceratitis* spp. [45] complexes. In the *B. tryoni* complex, the use of the COI gene as a DNA barcode is complicated by the presence of a pseudogene (a nuclear copy of the gene; “numt”); however a large deletion in the numt pseudogene enables the use of a modified primer to specifically amplify the mitochondrial copy [44]. Recently, COI barcoding has been used to investigate the population structure and invasion history of the melon fly, *Bactrocera cucurbitae* (Coquillett) in Asia with results suggesting that this pest spread from west to east and recently invaded China via the Yunnan province [57]. 

In the *Ceratitis* spp. complex, COI barcodes were able to discriminate the vast majority of species, with the exception of *C. capitata* (medfly) and *C. caetrata* (Munro) [45]. Identification of *C. cosyra* (Walker) was also complicated by the presence of a high level of intra-species variation in the COI gene due to the presence of multiple gene copies; however dividing these sequences into genetic clusters assisted in generating sufficient barcode differences for species discrimination [45].

While there has been a rapid increase in the use of COI barcoding for species identification and differentiation, there has been some concern regarding the concept of a single gene region being used to define all animal species [58,59,60]. Although mitochondrial genes such as COI are often considered preferable to nuclear genes due to a lack of introns and a reduced exposure to recombination events [61], they can at times prove problematic due to the maternal inheritance of these genes [61]. The concept that every species has a fixed character or barcode is somewhat misleading in an evolutionary sense, as it does not account for temporal variation and selection pressure [58]. The latter point is exemplified by the relatively rapid sequence divergence that can occur amongst allopatric (geographically separated) populations. Indeed, there are several disadvantages to COI barcoding (Table 1) including a potential loss of discrimination in cases of very similar, but nonetheless polyphyletic species, due to the high level of sequence conservation within the barcode region. In these instances, the use of multiple markers for DNA barcoding can enhance species discrimination. In two studies of tropical tachinid parasitoids, concurrent taxonomic analyses of the COI barcoding region and the 28S D2 and ITS-1 gene regions revealed that many of these parasitoids, previously thought to be generalists, are actually cryptic host specialists [52,53].

**Table 1:** Comparison of molecular techniques for tephritid and parasitoid detection and differentiation.

Technique	Description	Advantages	Disadvantages	References
DNA barcoding	PCR amplification and sequencing of a genetic marker (usually the mitochondrial COI gene)	Widely used in arthropod identification and the barcode database for tephritids in particular is growing rapidly due to the tephritid barcoding initiative Generic primers available for COI barcode region COI is generally useful for distinguishing closely related and less closely related taxa Alternate markers can be sequenced if COI barcode is not differential May be useful for taxonomic analyses May be useful for taxonomic analyses	Requires a large database of sequences for comparison Prior knowledge of the barcoding region is required when applied diagnostically Individual sequences may not provide sufficient discrimination when studying cryptic species complexes COI and other mitochondrial genes are maternally inherited which may result in decreased barcode diversity and skew phylogenetic inferences	[13,44,45,46]
Specific PCR	Targeted assay giving a presence/absence result for a particular genus or species	Useful diagnostically as it targets a specific taxon Can be used to target a specific genus, species or strain within a mixed sample (e.g., detection of parasitoid DNA within fruit fly larvae) No sequencing of the PCR product is required	Requires specific primer design, assay optimization and specificity testing prior to use as a diagnostic	[48,62,63,64]
Size differential PCR	Employs generic PCR primers but yields amplicons that differ in length. Usually targets the intergenic transcribed spacer regions (ITS)	Can discriminate between a range of species simultaneously Differentiation is by electrophoresis, no sequencing or other downstream processing of the amplicon is required	Size of amplicon needs to vary substantially to enable discrimination ITS regions contain repetitive regions that can result in PCR products with multiple bands	[39]
PCR-RFLP	Involves discrimination of species based on restriction profile of amplicons.	Can discriminate between a range of species simultaneously Can be used on a range of genetic markers (*i.e.*,: not restricted to size variable markers) Can provide an additional level of discrimination if differentiation based on size fails May be able to detect new types in some instances	Requires downstream digestion of amplified DNA Mutations may occasionally result in unidentified RFLP patterns	[13,48,65,66,67]
Multiplex PCR	Combines multiple primer sets with different specificities in a single assay	Detects and differentiates multiple species in a single assay Can be used on multiple genetic markers Discrimination is by electrophoretic size differentiation, so no downstream processing of amplicons is required Useful for simultaneous detection of species in mixed samples (e.g., detection of host and parasitoid DNA in one assay).	Can be difficult assays to optimise due to the presence of multiple primer sets Potential cross-hybridisation of primers may interfere with reaction	[68,69,70,71]
RAPD	Uses random primers to generate multiple PCR products resulting in a fingerprint for a particular species	Simultaneously targets multiple genetic loci and is therefore more useful for discriminating closely related or cryptic species DNA fingerprint is generated in a single reaction Data may be used for phylogenetic reconstruction in some instances	Some issues with reproducibility Can’t be used on mixed samples Only useful as a diagnostic if the RAPD fingerprint of the unknown specimen has already been resolved for comparison	[72,73,74,75,76,77]
AFLP	Involves ligation of adaptors to digested DNA followed by PCR amplification using primers that are partially adaptor and partially gene-specific	Simultaneously targets multiple genetic loci and is therefore more useful for discriminating closely related or cryptic species Very sensitive and more robust than RAPD Data may be used for phylogenetic reconstruction in some instances	Requires manipulations in addition to PCR Can’t be used on mixed samples Only useful as a diagnostic if the AFLP fingerprint of the unknown specimen has already been resolved for comparison	[78,79]
Microsatellite analysis	Involves PCR amplification of multiple reiterated repeat-containing loci that are hypervariable due to slipped-strand mispairing mutations	Simultaneously targets multiple genetic loci and is therefore more useful for discriminating closely related or cryptic species. Particularly useful for tracing populations When fluorescent primers are used, fragment analysis is readily automated Assays can be multiplexed during PCR and detection (fragment analysis) phases Some microsatellite assays can be applied across a number of different species	Assay development is time consuming initially Can’t be used on mixed samples	[32,80,81,82,83,84,85,86,87,88,89,90,91,92,93,94,95]
Quantitative PCR	Short regions of DNA are PCR amplified and products are detected either with SYBR green (double stranded DNA dye) or via specific probes labeled with fluorescent dyes	Amplification is monitored in real-time against standards of known concentration allowing for quantification of target DNA When using specific probes for amplicon detection, the reaction can be multiplexed for simultaneous detection of up to 4 or 5 species and can be used on mixed samples No electrophoresis is required, detection is automated and involves detection of fluorescence intensity Allows for rapid and high throughput detection	Specialised equipment required Multiplexing is limited by choice of fluorescent dyes	[96,97]
LAMP	Employs 3 sets of specific primers used for amplification under isothermal conditions. Yields a ladder of amplicons on electrophoresis or amplicons can be detected using SYBR green	Rapid and specific amplification under isothermal conditions Does not require specialized equipment when paired with SYBR green detection Technique is potentially the most suitable for field conditions Can be used with mixed samples due to primer specificity	Assays have a relatively complex design Only suitable for field conditions when paired with a simple DNA extraction method	[98,99]

The mitochondrial gene *cyt b* gene, encoding cytochrome B, has been less widely used but successfully employed to differentiate species such as those within the neotropical parasitoid genus *Notiospathius* [50]. In that study, relationships inferred from the *cyt b* gene were more closely correlated with morphological data compared to those from the COI gene. In aphid parasitoids (Aphidiinae), the mitochondrial 16S rRNA (mt 16S rRNA) gene [100] and the nuclear long wavelength rhodopsin (LWRh) gene [51,100] have recently been assessed as markers for barcoding individual species. The LWRh gene was able to discriminate between two species that the COI gene was unable to discriminate [51], while the mt16S rRNA gene was found to be a sensitive marker for early detection of immature parasitoids within their aphid hosts [100]. The mt16S rRNA gene has also been used recently to complement taxonomic analysis of the COI gene in elucidating relationships between populations of *Bactrocera caudata* (Fabricius) tephritids [101]; however in general mt16S rRNA and LWRh genes are not yet widely applied to tephritid species and their parasitoids. Nonetheless they may represent alternative molecular barcodes for taxa where the use of the COI gene is problematic. 

COI gene sequence information for fruit flies and their parasitoids has increased markedly in recent years, with barcodes now available for species within genera of commercial significance. Major fruit fly genera with barcoded representatives include *Ceratitis* [45,102], *Bactrocera* [43,44,101], *Dacus* [103], and *Anastrepha* [104]. Furthermore, the Tephritid Barcoding Initiative [105] aims to barcode approximately 2,000 tephritid species within a 2-year period. Major parasitoid genera with sequenced representatives include *Psyttalia* [13], *Diachasmimorpha* [48,106] and *Fopius* [48].

### 2.2.PCR-based Approaches: PCR-RFLP, Multiplex PCR, RAPD and AFLP

Other PCR-based methodologies for species detection and discrimination differing from barcoding procedures in that they often employ primer sets specific for the species or genus under investigation. PCR primers with a high degree of specificity make downstream sequencing of the amplicon unnecessary because the presence/absence of an amplicon, as confirmed by electrophoresis, indicates whether an insect belongs to the target genus or species. 

In some cases, species can be differentiated based on electrophoretic analysis of amplicon size where primers are designed to target sites adjacent to genome regions that vary in length between species. The ITS regions have been used for this purpose as they tend to be quite variable in length as well as sequence, while the highly conserved ribosomal RNA genes flanking the ITS regions can be exploited as primer target sites to ensure successful amplification [67]. However, most genes vary only in sequence but not in length, or the length may not vary sufficiently to allow for electrophoretic discrimination. In these cases, PCR products can be subjected to digestion with restriction enzymes generating restriction fragment length polymorphisms (PCR-RFLP). This method relies on the fact that restriction enzymes cleave DNA at specific sequences; thus variation in sequences amongst species can be exploited to generate DNA fragments that vary in size, which are then detectable by electrophoresis. Sibling species of the lepidopteran parasitoid, *Trichogramma* can be successfully distinguished in this manner, using variation in the length of the ITS-2 region and PCR-RFLP of the same region where the sizes of the PCR products are similar [39]. A similar method was attempted for discrimination of tephritid parasitoids of the genera *Psyttalia* [13] and *Diachasmimorpha* [48]; however the presence of single nucleotide repeats in these gene regions appears to compromise the quality of the resultant PCR products, suggesting that the ITS gene regions may not be the most suitable loci for discrimination of certain fruit fly parasitoids. In the case of members of the genus *Psyttalia*, aPCR-RFLP assay based on digestion of the COI barcode region was proposed for discrimination of these species [13]. A similar COI PCR-RFLP assay has been designed and tested on key parasitoids of the Queensland fruit fly, *Bactrocera tryoni* and successfully discriminates between *D. longicaudata*, *D. tryoni*, *D. kraussii* and *Fopius arisanus* (Sonan) [48].

PCR-RFLP has also been used successfully for molecular diagnostics of tephritids. Barr *et al*. [66] targeted three mitochondrial genes (12S and 16S rDNA and NADH-dehydrogenase subunit 6) for PCR-RFLP analysis of *Ceratitis* spp. and were successfully able to discriminate 25 species and two species clusters. Three of the remaining five species that could not be distinguished were resolved by analysis of ITS-1 amplicon lengths. 

Fragment length polymorphisms can also be generated using random PCR primers (random amplified polymorphic DNA, RAPD) or primers targeting adaptors ligated to restricted DNA in a technique called amplified fragment length polymorphism (AFLP) [107]. With both methods, each specimen produces a DNA fingerprint, representing multiple loci that can be compared to assess genetic similarity. RAPD analysis has been used for the genetic differentiation of a number of different tephritid populations. Baruffi *et al*. [72] used RAPD fingerprints to examine genetic differences between wild and laboratory populations of *C. capitata*, with African populations showing increased intraspecific variability compared to laboratory populations [72]. More recently the RAPD typing technique has been used to demonstrate genetic variability in Moroccan *C. capitata* populations from different altitudes [73]. In another study, RAPD data was combined with data from alternative typing methods to demonstrate that *C. capitata* from different geographic regions displayed considerable genetic variability and used this information to reconstruct the evolutionary history of the spread of *C. capitata* across the globe from Africa to Europe, Hawaii and Central America, with secondary colonisation occurring in Australia from European populations [74]. RAPD analysis has also been used for the differentiation of immature (and morphologically similar) forms of tephritids where their ranges overlap, such as *C. capitata* and *Anastrepha fraterculus* (Wiedemann) in Argentina [75] and *C. capitata* and the guava fly, *Bactrocera zonata* (Saunders), in Egypt [76]. 

RAPD markers have only occasionally been used for discrimination of parasitoids. In a study by Karam *et al*., RAPD analysis demonstrated genetic differences between wild and mass-reared strains of *P. concolor* (Szèpligeti) from the Middle East, Mediterranean and Guatemala [77]. Specific PCR primers for differentiation of species were then developed based on key RAPD loci with the aim of using these assays on field-released strains. AFLP has also been used to study allopatric populations of *Psyttalia* spp. in Africa and along with morphological data this demonstrated that some populations belonged to the *P. concolor* group, while another belonged to the *P. perproximus* (Silvestri) group [78]. This method has also been employed to identify species-specific markers in the tephritids *C. capitata* and *C. rosa* (Karsch) [79]. Despite the fact that AFLP is considered by many to be a more reproducible and reliable method than RAPD for fingerprinting species, it has been relatively under-utilised in the study of fruit flies and their parasitoids. Nonetheless both of these techniques are limited to DNA samples that are derived from a single source. Consequently, these methods could not be used for identification of parasitoid DNA within host larvae as this would confound the DNA fingerprint. 

Multiplex PCR assays, whereby two or more specific primer sets are included in a single reaction [108], are useful where detection and differentiation of a number of different species is required. In multiplex PCR, primer sets must be designed such that there is sufficient variation in the resulting amplicons that they can be clearly separated via electrophoresis. Multiplex PCRs are more difficult to design than standard singleplex assays due to the increased possibility of primer-primer interactions which can result in failure to amplify the target gene regions. However, multiplex PCR can be an efficient tool for species identification as it bypasses the need for downstream manipulations such as restriction digestions [108]. A multiplex method based on the ITS gene regions has been used successfully for differentiation of *Persistenus* parasitoids of *Lygus* bugs (Hemiptera: Miridae) and also for detection of these parasitoids within host nymphs [68]. Multiplex PCRs have also been applied to the study of trophic interactions in host-parasitoid-predator systems [69,70,71] by analysis of predator gut contents. One of these studies showed that generalist predators of aphids also directly preyed on adult aphid parasitoids, thereby attenuating overall pest suppression [70]. While multiplex PCR assays may have similar applications in the study of tephritid pests and their parasitoids, they have not yet been applied for this purpose, although they are routinely used for analysis of microsatellite loci (see later). 

Applications of PCR technology to fruit fly parasitoid augmentative release programs are also promising, particularly for the detection of parasitoid DNA (eggs) within host larvae. Molecular approaches for within-host detection have been used as a diagnostic for Hymenopteran parasitoids of *Lygus* bugs [63,64,65], cotton bollworm [62], cereal aphids [109], the brown citrus aphid [110], white flies [71], house and stable flies [67] and the European corn borer [111]. Where PCR is used to detect parasitoid DNA within a host species, careful consideration must be given to potential cross-reactivity of primers with host DNA and to the fact that multiple parasitoid species may parasitise the same host. In several of these studies, specific PCR primer sets [62,63,110,112] or PCR followed by RFLP analyses [65,67] were able to differentiate as well as detect individual parasitoid species within the host larval, pupal or egg phases. In most instances, parasitoids can be detected within 24 h of oviposition [62,63,64,110].

While within-host molecular detection of parasitoids has been applied to a wide range of pest species, only a single study has applied this technology to fruit fly parasitoids. COI barcode sequences for the *Diachasmimorpha* species, *D. tryoni*, *D. kraussii* and *D. longicaudata* as well as *F. arisanus* have recently been elucidated, enabling the development of a PCR-RFLP based on the COI gene for detection and differentiation of these species within *B. tryoni* [48]. Parasitoids of the genera *Diachasmimorpha* and *Fopius* are widely used for augmentative release against a variety of fruit fly pests and therefore this assay may be of significant benefit to biological control programs worldwide.

Careful PCR assay design has enabled innovative applications for other fruit fly control strategies. One ingenious study utilised a specific PCR assay coupled with a PCR-RFLP assay to assess mating success of sterile *C. capitata* [113]. The PCR assay, specific for the *C. capitata* Y chromosome, was used to detect sperm within the spermathecae of mated female insects, while the PCR-RFLP aimed to discriminate between mitochondrial DNA from a wildtype strain and that of the Vienna 8 strain of *C. capitata*, used for sterile release. Application of these molecular techniques in the context of SIT programs would bypass the reliance on less precise estimates of mating success such as the use of egg hatchability or spermatozoid head size measurement [113]. 

### 2.3. Microsatellite Markers

Microsatellites refer to polymorphic regions within a genome composed of short tandem nucleotide repeats (usually 2–7 base pairs in length) [114]. Mutation occurs more frequently in repetitive DNA, including microsatellites, due to a phenomenon known as slipped-strand mispairing. Slipped-strand mispairing occurs during DNA replication resulting in the loss or addition of an entire repeating unit, or several repeating units, contributing to polymorphism at that locus [115]. Indeed, the number of individual repeating units in microsatellite regions may range from a few to 50 or more, resulting in alleles that are highly variable in length [114]. 

**Figure 1:**
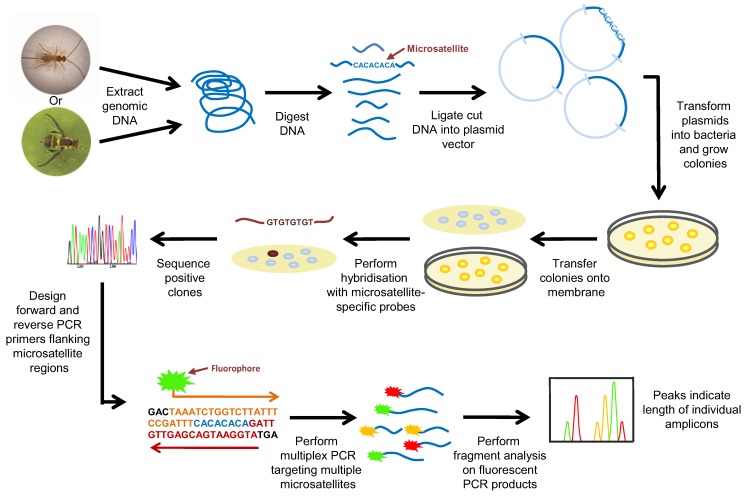
Development and use of microsatellite markers for species identification.

Microsatellites occur at numerous loci within genomes and several of these markers are usually examined simultaneously to generate a fingerprint for a given specimen [114]. Appropriate microsatellite markers were originally identified by screening a genomic clone library with probes complementary to commonly occurring repeat regions (Figure 1). Clones that hybridise to the probe/s are then sequenced to characterise the microsatellite loci and the adjacent DNA sequences. Once appropriate microsatellite markers have been identified, primers that target the specific regions flanking the microsatellite loci are used for PCR amplification. To facilitate DNA fragment analysis, one primer used in the PCR is labelled, typically with a radioactive or fluorescent tag; the optimal length for PCR amplicons is in the range of 100–200 bp. Following amplification, discrimination of these loci is performed by electrophoresis on a polyacrylamide gel which allows for sufficient size resolution of the fragments. Most modern microsatellite analyses are usually carried out with fluorescently-labelled primers, and an automated genotyper is used for fragment analysis (DNA separation and fluorophor detection) (Figure 1). In many instances, microsatellite PCRs are multiplexed and primers are labelled with different fluorophores to allow for more rapid and efficient genetic analysis.

With advancing technology, the techniques used to identify microsatellites has evolved. Currently three methods exist: (1) the traditional method of screening a genomic clone library with probes complementary to commonly occurring repeat regions as previously described; (2) an automated method where bioinformatics software is used to search publically available databases for microsatellite markers, also referred to as data mining; and (3) next generation sequencing for the identification and screening of microsatellites [116]. Data mining through *in silico* analysis of publically available databases is an efficient and cost effective method for the identification of microsatellite loci. There are a number of tools including Basic Local Alignment Search Tool for nucleotides (BLASTN) that compares a given nucleotide query sequence with sequences contained in a nucleotide data base [117], MIcroSAtellite (MISA), which is a microsatellite mining tool [118] and Tandem-Repeats Finder [119] with other tools including SciRoKo [120] which is suitable for whole genome sequence mining. These and other tools are extensively covered in a review by Sharma *et al*. [121]. Next generation sequencing is revolutionizing molecular ecology with the reduce costs and the simplified means of identifying suitable molecular genetic markers such as microsatellites [122]. With increased accuracy and read lengths this data is suitable for microsatellite development. Next generation sequencing allows for a higher number of microsatellite loci to be identified at a reduced cost, which in turn allows for higher stringency in the selection of the appropriate loci. Guichous *et al.* 2011 [123] has provided a review on the current trends in microsatellite genotyping including the use of multiplexing, while Gardner *et al*. 2011 [122] has provided a workflow process for next generation sequencing development of microsatellites.

Because microsatellites display a high level of polymorphism both between and within species, and are typically co-dominant, they are particularly useful for studying cryptic species complexes and population genetics. Indeed, microsatellites markers have been widely used in the study of tephritid populations and have revealed valuable information regarding the spread of these pests. Microsatellites identified in the Queensland fruit fly, *B. tryoni* [80] were used to demonstrate that populations of this species within five separate regions of eastern and central Australia remained relatively homogenous over a five year period [81]. While results indicated that little or no differentiation was occurring in the core population of flies in the north east of Australia, probably due to the capacity of *B. tryoni* for dispersal, significant heterogeneity was observed between the core population and satellite (south eastern and central Australian) populations of this species. Thus, while the mobility of *B. tryoni* overcomes any tendency for temporal heterogeneity within the core population, migrating flies had little impact on microsatellite patterns in other geographic populations. The divergence between *B. tryoni* populations was partially attributed to adaptation of this species to the varying climactic conditions within these different geographic regions. The finding that these populations are relatively static is promising for the success of future programs aimed at targeted biological control of *B. tryoni* within these separate regions, given that flies located in warmer climates are not adapted to cooler climates, thus limiting their spread [81]. A more recent study on *B. tryoni* in Australia used microsatellites to demonstrate that at the southern limit of its range, the fruit flies form genetically differentiated source populations [82]. This region is an important horticultural area, and therefore this study has significant implications for fruit fly management programs in the region, particularly those involving SIT or augmentative release. Indeed, determining the source of a fruit fly incursion enables the implementation of targeted management strategies.

Microsatellites have also been characterised for other tephritids and have been used to study the population structure of *B. dorsalis* in Taiwan [83], *B. oleae* in California [84] and *B. cucurbitae* in south east China [85]. Interestingly, analysis of seven microsatellite markers [86] in *A. suspensa* populations in Florida and the Caribbean indicated that continuous gene flow between fruit flies attacking different host plants and from different geographic locations has resulted in low genetic diversity between populations [87]. These results contradicted prior anecdotal descriptions of regional variation in fruit preferences, but are in agreement with molecular analyses of the COI gene which also indicate that populations are homogenous [87]. 

Microsatellite markers have been analysed in several biological invasion studies to trace a putative origin for some tephritid pests. Zygouridis *et al*. [84] employed 10 microsatellite loci, previously characterised for study of *B. oleae* in Europe [88,89], to demonstrate that olive flies in California most probably derived from east Mediterranean populations. Similarly, microsatellite analyses complemented data from alternate molecular markers in demonstrating that infestations of *C. capitata* in Florida in the 1990s could be traced to flies of Mediterranean origin [90]. Baliraine *et al*. [91] found that a proportion of microsatellite PCRs developed for *C. capitata* can also be applied to the analysis of *C. rosa*, *C. cosyra* and *C. fasciventris* (Bezzi). Furthermore, differential presence or absence of a subset of these loci indicated that they may prove useful for discrimination of these species, expanding the applicability of this assay to other pest species. Similarly, around 50% of microsatellite assays developed for *B. oleae* alsoexhibit cross-species amplification in other *Bactrocera* spp., in *Ceratitis* spp. (35%), *Rhagoletis* and *Anastrepha* spp. (28 and 24% respectively) [32].

Microsatellite analysis is a powerful tool that can also be applied to the characterisation of fruit fly parasitoid populations; however researchers have been slower to adapt the technology for this purpose. However, a number of microsatellite markers have recently been identified for braconid parasitoids including *Diachasma alloeum* (Muesebeck), which parasitises the apple maggot fly, *Rhagoletis pomonella* (Walsh) [92], *Diachasmimorpha tryoni* [93] and African populations of the olive fly parasitoid, *Psyttalia lounsburyi* (Silvestri) [94]. Assays amplifying 21 microsatellite loci from *P. lounsburyi* were developed and multiplexed [94] for ongoing monitoring of a field-released strain of this parasitoid in a biological control program for olive fly in southern France [38]. Members of two African populations (Kenya and South Africa) of *P. lounsburyi* that are genetically distinct according to microsatellite patterns, along with a hybrid of these two strains, were released into 60 field sites in 2008. Microsatellite studies aimed at assessing the success of introduced *P. lounsburyi* parasitoids against olive fly over time, and to determine whether hybridisation of the two geographical strains affects the establishment and growth of the parasitoid population [38], are continuing. 

Several studies have examined the potential for cross-species amplification of microsatellites thereby expanding the potential utility of these assays in parasitoid population analyses [92,94]. Indeed, many of the *P. lounsburyi* microsatellite assays developed by Bon *et al*. [94] showed cross-reactivity with an African strain of *P. concolor* and a south central Asian strain of *P. ponerophaga* (Silvestri)*. P. concolor* parasitises a wide range of tephritids in some major pest genera including *Anastrepha*, *Bactrocera*, *Ceratitis* and *Dacus* [10] and has been used in augmentative control of *C. capitata* and *B. oleae* [13]. Therefore, these microsatellite markers may also prove useful for monitoring the success of biological control programs involving release of *P. concolor.* Microsatellite markers developed for the apple maggot parasitoid, *Diachasma alloeum*, alsoshowed cross-reactivity with another apple maggot parasitoid, *Diachasmimorpha mellea* (Gahan), as well as a parasitoid of the cherry fruit fly (*Rhagoletis cingulata* (Loew)), *Diachasma ferrugineum* (Gahan), indicating that they could be broadly applied to future biological control programs involving these species [92]. Interestingly, microsatellite analysis, along with limited data from COI barcoding, demonstrated that *D. alloeum* parasitoids have co-speciated with *Rhagoletis pomonella* siblings that are phytophagous on different plant species [95].

### 2.4. New Technologies

Worthy of note are frontier molecular technologies that have been developed with recent or potential applications to the study of fruit fly-parasitoid interactions. Quantitative PCR (qPCR) technology has a number of advantages over conventional PCR including decreased sample handling due to the fact that electrophoretic analysis of PCR products is unnecessary. This technology affords rapid sample analysis with a potential for high throughput. Detection of PCR products occurs either via a SYBR-green dye within the reaction mix that fluoresces in the presence of double stranded DNA (amplicon) but not single-stranded DNA, or via a specific, fluorescently-labelled probe specific for the amplicon being detected. The latter method is particularly useful where multiplexing is desirable since numerous probes labelled with different fluorophores can be used for detection simultaneously. To date, qPCR assays have only been developed for the identification of *Bactrocera latifrons* (Hendel) [96] as well as *B. philippinensis* (Drew & Hancock) and *B. occipitalis * (Bezzi) [97]. In the latter study, a SYBR-green assay was employed and amplicon differentiation was possible by analysing the melt curves of the products [97]. Another major advantage of qPCR technology over conventional PCR is the ability to quantify target DNA present within a sample. This is a feature that would be particularly useful for within-host detection of parasitoids as it may be capable of providing data on super-parasitism; however no studies to date have developed qPCR assays for fruit fly parasitoid detection.

Loop-mediated isothermal amplification (LAMP) is another form of DNA amplification that has been developed relatively recently [98]. This method employs three specifically designed pairs of primers and is carried out under isothermal (constant temperature) conditions. LAMP produces large quantitites of amplicon relatively quickly and the products vary in size so they appear as a ladder in electrophoretic analyses; however detection is often via the addition of SYBR-green or similar dyes to avoid downstream manipulations. Indeed, the major advantage of LAMP technology is that it does not require specialised equipment and can be performed using a simple heating block, so it is considered a useful method for identification of specimens in the field. Huang *et al*. [99] recently developed a LAMP assay for *C. capitata* at different stages of development; coupled with simple DNA extraction procedures and SYBR-green dye, the authors were able to specifically identify *C. capitata* flies within 1 h. LAMP assays have not yet been developed for fruit fly parasitoids, but would be an extremely valuable tool in evaluating augmentative control programs by enabling rapid detection of parasitism in the field.

Another new technology is Restriction site Associated DNA (RAD) commonly referred to as RADtag or RADseq this technique capitalises on restriction sites that are located throughout the entire genome, with different restriction enzymes generating different RADtag densities [124]. This technique involves digesting the target DNA with the selected restriction enzyme. Biotinylated adapters are ligated to the overhangs and the DNA is sheared into smaller fragments. RADtags are purified using streptavidin beads to isolate the biotinylated fragments. Digestion with the same restriction enzyme causes the release of the DNA from the beads, which are then detected by differential hybridization patterns on a microarray [124]. This technique has been used successfully to determine genotypes in adult *Drosophila melanogaster* Meigan, 1830 (Diptera: Drosophilidae) [124]. Double adaptor RADtag has also been used where a second adaptor is ligated and PCR is used to amplify the fragments that contain both adaptors. In this method the first adaptor contains a barcode for sample tracking. High-throughput sequencing is then used to analyze the RADtags [125,126]. This method has recently progressed into double digest RADseq, which combines laboratory and computation methodology permitting greater flexibility and robustness in region recovery. It is also compatible with a microplate format to reduce both time and costs compared to traditional RADtag methodologies [127]. This methodology has the potential to be applied to diversity of biological questions from genotyping and biological diversity studies in both fruit flies and parasitoids [128].

With the advent of efficient and inexpensive new sequencing technologies, genomic approaches including whole genome sequencing are becoming more common. Whole genome sequences are not currently available for tephritids or their parasitoids, although several tephritid genome projects are planned [127]. A number of complete tephritid mitochondrial genomes have been sequenced including that of *C. capitata* [129], *B. oleae* [130] and *B. dorsalis* [131], and transcriptome studies of the apple maggot fly, *Rhagoletis pomonella* are also underway [132]. In addition, >20,000 expressed sequenced tags have been identified in *C. capitata* and these will be invaluable for research into *C. capitata* biology and the development of new techniques for control of this widespread pest [133]. While parasitoids of tephritids have not yet been subjected to genome-based studies, the recent sequencing of three species of the parasitoid wasp genus, *Nasonia* (Hymenoptera: Pteromalidae) provided interesting insights into the mode of action of these species [134]. Most notably, *Nasonia* spp. were found to possess venom composed of a diverse array of unique proteins responsible for physiological changes in the host such as immune suppression, alteration of behaviour, developmental arrest and apoptosis [135] and a locus responsible for determining host preference [134]. Thus, genome sequencing of the key fruit fly parasitoids, perhaps paired with transcriptome studies of parasitised host species, would be highly desirable to assist in the identification and characterization of attributes of parasitoid strains relevant to effective augmentative control.

**Figure 2:**
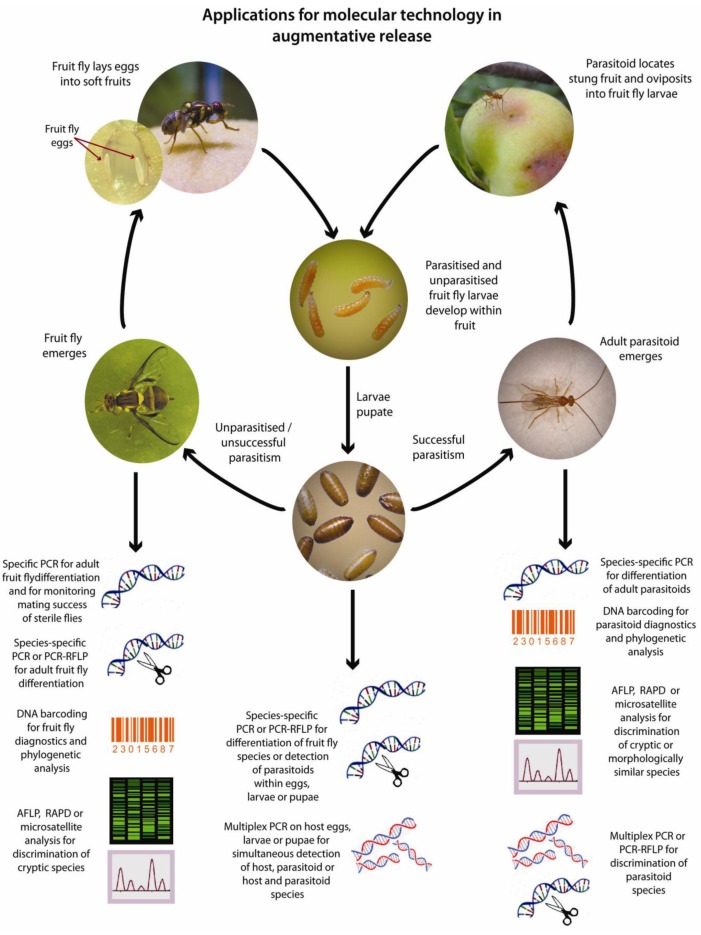
Applications for molecular techniques in the augmentative release of parasitoid wasps against tephritids. (Photographs by Max Hill and Lowan Turton, modified with permission ©NSW DPI).

## 3. Conclusions and Implications of Molecular Technology for Augmentative Fruit Fly Control Programs

Successful augmentative fruit fly control programs rely on prior knowledge of the tephritid population structure as well as parasitoid identity and host preferences [136]. The ability to trace host and parasitoid movements is also a significant advantage in monitoring the success of a given program [38]. Traditional approaches relying on phenotypic and morphological data to identify species may be adequate where species possess clearly defined structures and where specialist knowledge is at hand, however these are often not available. Several of the major tephritid pests belong to cryptic species complexes making morphological discrimination of adult forms difficult or impossible (species are sometimes identified based on their geographic location rather than morphology) and similar issues also exist in some parasitoid genera [13]. Relying on morphological data is even more problematic when attempting to identify the larval forms of tephritid flies that may lack distinctive phenotypic features or indeed the immature stages of braconid parasitoids which are endoparasitic. Furthermore, traditional methods for identification of parasitoids within hosts and estimations of parasitism rate rely on laborious and lengthy rearing and dissection processes (average 5–6 weeks from egg to adult depending upon fly/parasitoid/host plant) making molecular detection appealing where a rapid result is required. Indeed, PCR-based studies conducted on arthropod parasitoids indicate that these can be detected within host larvae or eggs from samples collected within 24 h of oviposition [62,63,64,110]. 

There are several stages within the fruit fly and parasitoid lifecycles where molecular techniques are being, or can be applied (Figure 2). Despite clear advantages in the use of these technologies, molecular methods are not yet widely applied to monitoring the success of augmentative tephritid control programs. Such molecular tools are however becoming increasingly important in clarifying relationships between species, strains and populations of both fruit flies and their parasitoids, which are critical steps in planning successful releases. Indeed, these recent advances are likely to increase the focus on augmentative methods for fruit fly control in the future.
